# Short-term visual deprivation boosts the flexibility of body representation

**DOI:** 10.1038/s41598-018-24496-8

**Published:** 2018-04-19

**Authors:** Dominika Radziun, H. Henrik Ehrsson

**Affiliations:** Department of Neuroscience, Karolinska Institutet, Stockholm, Sweden

## Abstract

Short-term visual deprivation by blindfolding influences tactile acuity and orientation in space and, on a neural level, leads to enhanced excitability of visual and motor cortices. However, to the best of our knowledge, the possible effects of short-term visual deprivation on body representation have not been examined. In the present study, we tested two groups of 30 healthy participants with the somatic rubber hand illusion, a well-established paradigm to probe the dynamic plasticity of body representation. Before the start of the procedure, the experimental group was blindfolded for 120 minutes, while the control group wore transparent goggles for the same amount of time. We found that although there was no difference in the subjective feeling of ownership of the rubber hand during the illusion, the blindfolded group showed a significantly larger recalibration of hand position sense towards the location of the rubber hand than the control group. This finding suggests that short-term visual deprivation boosts plasticity of body representation in terms of multisensory spatial recalibration of hand position sense.

## Introduction

The self-attribution of body parts depends on the integration of information from different sensory modalities, including vision, touch and proprioception^1–6^; for a review, see^7^. Vision often plays a leading role in this integration process, since it often dominates other modalities under good viewing conditions due to its high spatial resolution and good signal-to-noise ratio^8–15^; but see also^16^. Research on people with visual impairment – both congenital and acquired – can be helpful to understand the functional, developmental, and neuroanatomical role of vision in body representation. Crucially, visual information seems to be important for the formation of external spatial reference frames centered on the body, which facilitate the integration of visual and tactile signals in a common coordinate system^4,5,17,18^. In sighted individuals, tactile signals are automatically remapped into an external coordinate system, which presumably requires previous visual experience. Whether this body-centered external spatial reference frame is innately determined or acquired during development, is still an open question but empirical evidence from audio-tactile interaction tasks suggests that early sensory experience plays a crucial role in its formation^19–22^.

One of the paradigms that require remapping touch into an external spatial reference frame for fusion with visual signals is the rubber hand illusion^1^. The “classic” version of this illusion is elicited by simultaneously brushing the participant’s real hand, which is hidden behind a screen, and a life-sized rubber hand in full view of the subject. A relatively short period (approximately 10–20 s – see^3,23,24^ of such repeated visuo-tactile stimulation elicits a perceptual illusion that the touches one is seeing originate from the rubber hand and that this model hand is one’s own hand (“feeling of body ownership”)^3^. In the non-visual version of the rubber hand illusion – the so-called somatic rubber hand illusion^25^ – the experimenter moves the blindfolded participant’s left index finger so that it touches the knuckle of a rubber right hand while synchronously touching the participant’s right hand at the corresponding site. This bilateral tactile-proprioceptive stimulation produces a feeling of touching one’s own right hand directly with the left index finger, although one is in fact touching a cold and hard rubber hand. As suggested by Makin *et al.*^5^, external reference frames play an important role in the process of attributing body parts to the self in the rubber hand illusion paradigm. More specifically, the somatosensory signals are probably remapped into this common external coordinate system to facilitate visuo-proprioceptive integration^26^. On a neural level, both the “classic” and the “somatic” rubber hand illusions are associated with activity in multisensory areas in the frontal and parietal association cortices, most notably the ventral premotor cortex and intraparietal cortex^3^, which suggests that these areas contribute to the self-attribution of body parts by integrating visual, tactile and proprioceptive signals^27^.

Both somatic and “classic” rubber hand illusions can be quantified subjectively with questionnaires and objectively as changes in position sense of the real hand towards the location of the rubber hand, which is known as proprioceptive drift^1,4,25^. The questionnaires measure the subjective sense of ownership of the rubber hand, which presumably is related to the formation of a coherent multisensory representation of the rubber hand as one’s own^7^. In the somatic rubber hand illusion, this process corresponds to the participants’ high affirmative ratings on the statement “I felt as if I was touching my right hand with my left index finger” in a commonly used questionnaire^25^. Furthermore, the proprioceptive drift comes as a result of a process of multisensory spatial recalibration, during which the position sense is being dynamically updated to correspond better with the locations of the visual and/or tactile events in the external space. Although proprioceptive drift often correlates with body ownership in the rubber hand illusion experiments see^4,28–30^, the causal relationship between the two is unclear. In fact, a growing number of studies have shown that ownership and proprioceptive drift can deviate from each other under certain conditions^11,31–33^. Thus, the questionnaires and the proprioceptive drift probably register different aspects of the rubber hand illusion.

Because the somatic version of the rubber hand illusion does not require any visual input, it can be a useful tool to compare multisensory body representation in both blind and sighted individuals. The study of body ownership in blind individuals can provide important information about the role of long-term visual experience for the sense of bodily self. The first such study was conducted by Petkova and colleagues^34^, who compared one group of blind individuals who had had severe visual impairment or complete blindness from birth with a sighted control group. Interestingly, blind individuals did not show any signs of the illusion – they strongly denied feeling any ownership of the rubber hand and did not show a significant proprioceptive drift towards the model hand. These observations suggest that extensive visual experience is necessary to dynamically integrate tactile and proprioceptive information from the two hands and form the illusory multisensory percept of self-touch. However, this study does not tell us if the visual experience during development or the visual experience as an adult is the critical factor. To address this issue, Nava and colleagues^35^ compared two groups of congenitally blind and late-blind participants and tested them on the somatic rubber hand illusion to directly examine if there is an effect of developmental vision on body representation. Although both groups firmly rejected experiencing the illusion in the questionnaire and reported similar degrees of rejection, the late-blind participants showed significantly larger proprioceptive drift towards the rubber hand than did the congenitally blind. In fact, the proprioceptive drift in the late-blind group was similar in amplitude to that in the sighted control group. Thus, both congenitally blind and late-blind individuals have less flexible representation of their own body, which makes it difficult for them to experience the rubber hand illusion. Nevertheless, visual experience during development seems to be sufficient to obtain the ability to spatially recalibrate the position sense of the hand based on multimodal feedback, as the late-blind group demonstrated this ability.

Blindfolding is a classic experimental model system of blindness that is used to study the effects of deprived visual experience on various perceptual, spatial and cognitive abilities in sighted volunteers. Remarkably, as suggested by Merabet and colleagues^36^, the relatively fast plastic changes as a result of blindfolding can lead, if sustained and reinforced, to more permanent changes, very similar to those observed in the blind. Even short-term visual deprivation for periods of 90 to 120 minutes might produce detectable and relatively large improvements in tactile acuity^37,38^; but see also^39,40^ and sound localization^41^, sometimes with similar performance levels to those observed in early blind people^42^. This finding indicates that blindfolding can, to some extent, produce similar cognitive and perceptual effects as blindness. On a neural level, short-term visual deprivation for 30 to 180 minutes leads to enhanced excitability of visual and motor cortices^43–46^; but see also^47^, while more prolonged visual deprivation on the order of 5 days results in rapid, early plastic changes in the visual cortex^36^. Thus, an interesting question would be if there is any effect of short-term visual deprivation on the subjective feeling of limb ownership and the spatial recalibration of hand position sense in the somatic rubber hand illusion, i.e., if multisensory integration of bodily signals in a common external spatial reference frame would be diminished already after such a short period of visual deprivation.

To address this issue, we compared two groups of 30 subjects with the somatic rubber hand illusion. Before the start of the procedure, the experimental group was blindfolded for 120 minutes, while in the control group, the eye-covering black band was replaced with a transparent pair of glasses. Our hypothesis was that the short period of blindfolding would lead to distortions in body representation similar to those in late-blind subjects described above. Thus, we predicted that in the case of subjective measures of the illusion, the blindfolded participants will not experience that they were touching their own hand, or at least, this sensation should be significantly diminished, but in the case of the objective measure – proprioceptive drift – there should be no significant difference between the blindfolded group and the control group.

## Methods

### Subjects

We tested a total of 60 healthy volunteers: 30 in the blindfolded group (mean age = 29.3, range = 21–56; 20 females, 10 males; 28 right-handed, 2 left-handed) and 30 in the control group (mean age = 29.2, range = 19–52; 22 females, 8 males; 29 right-handed, 1 left-handed). The difference in age between the groups was not significant (t (58) = 0.047, p = 0.963). The sample size was based on previous experiments using the rubber hand illusion e.g.^48,49^. All participants were recruited through advertisements on the campus of the Karolinska Institute and on social media. None of them had prior experience with the somatic rubber hand illusion. Participants received 500 SEK (an equivalent of €50) as compensation. All subjects had normal or corrected-to-normal vision. The experiment was approved by the local ethical committee (Regional Ethical Review Board of Stockholm), and the experiment was carried out in accordance with the approved guidelines. A written informed consent form was obtained from each participant.

### Task and procedure

**Figure 1:**
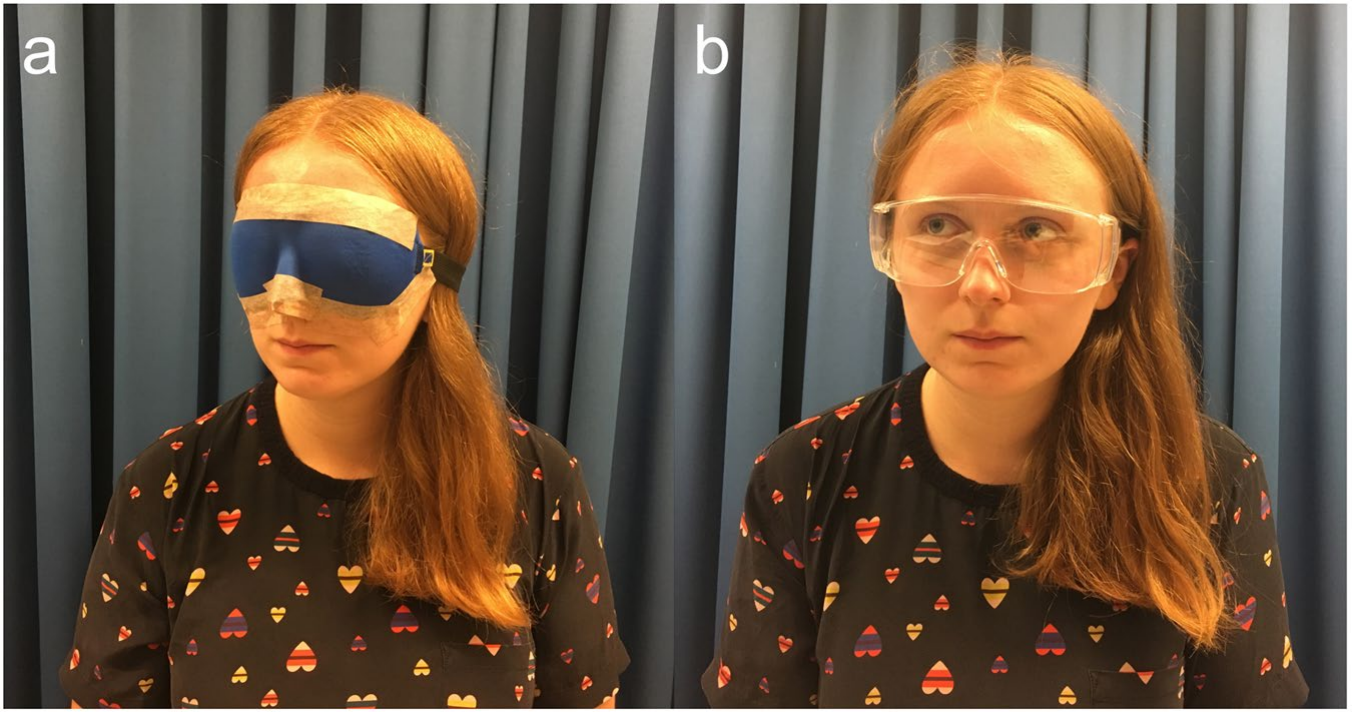
Short-term visual deprivation. The blindfold used in the study (**a**) and the transparent goggles worn by the participants in the control group (**b**).

Before the start of the experiment, all participants were informed about the experimental setup and received a short description of the procedure. Then, subjects from the experimental group were fully blindfolded for 120 minutes (the duration of blindfolding was based on previous short-term visual deprivation studies, e.g^38,39,44^. The blindfold prevented all light from reaching the eyes; medical tape was placed around it to avoid accidental displacement (Fig. 1a). The subjects in the control group wore a pair of transparent safety goggles, enabling them to see the environment without any restrictions (Fig. 1b). Then, all of the participants sat on a chair in a comfortable position and listened to a previously prepared playlist with music or podcasts using binaural headphones (MEL 1600530 by Maxell, Tokyo, Japan) in order to remain alert throughout the first part of the procedure before the rubber–hand illusion experiment commenced. Once every 30 minutes, the experimenter informed participants about the time left and made sure that they did not report any drowsiness. All of the participants remained alert for the whole duration of the experiment, as they reported to the experimenter who accompanied the participants during the whole procedure.

**Figure 2:**
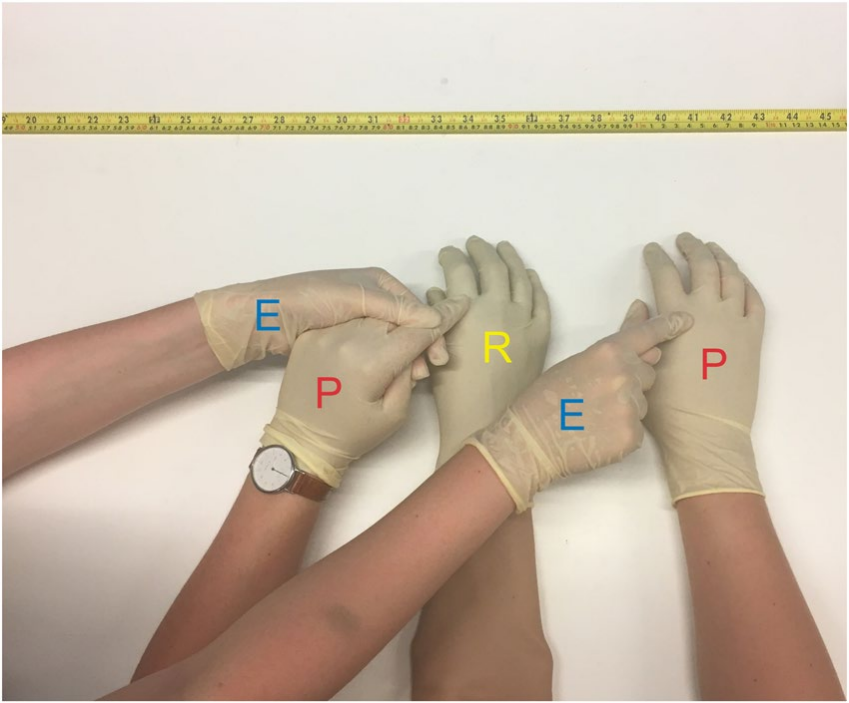
The illusion induction. The setup used to induce the somatic rubber hand illusion. From left to right: (i) the experimenter’s left hand holding the participant’s left index, (ii) the participant’s right hand, (iii) the rubber hand, (iv) the experimenter’s right hand touching the participant’s right hand with the index finger, (v) the participant’s right hand. At the top of the picture is the ruler used to measure the proprioceptive drift and confirm the 15-cm distance between the rubber and the participant’s hands. Synchronous touches on the rubber hand and the participant’s real right hand trigger the illusory self-touch. Each of the participant’s hands is marked with a red “P”, each of the experimenter’s hands with a blue “E” and the rubber hand with a yellow “R”.

After 2 hours, participants placed both of their hands in a relaxed position on a table in front of them, while a gender-matched rubber hand was placed between the right and left hands. The participants from the blindfolded group kept their blindfold on throughout the entire rubber hand illusion procedure. The participants in the control group wore a blindfold during the illusion test. Before the start of the illusion induction procedure, participants from both groups (both wearing blindfolds at this point) were allowed to tactilely explore the surface of the rubber hand, so that, at the outset of experiment, participants knew they were touching a rubber hand. The experimenter, the participant and the rubber hand all wore identical plastic surgical gloves to make the tactile surfaces of the hands as similar as possible. The distance between the participant’s right index finger and the index finger of the right rubber hand was always 15 centimeters. The experimenter moved the participant’s left index finger so that it touched the knuckle of the rubber hand’s index finger (Fig. 2). At the same time, the experimenter touched the knuckle of the right index finger of the participant. The period between the taps was approximately one second and each tap lasted approximately 300 ms. The taps were applied with the following pattern: three taps, one-second break, two taps, one-second break, and so on. Each touching session lasted approximately 60 seconds, so that one trial consisted of approximately 46 taps.

The experiment consisted of eight semi-randomized trials. First, six localization trials were conducted (three blocks of two trials) to measure proprioceptive drift, and second, two trials were conducted for the questionnaire. The proprioceptive drift trials were always performed before the questionnaire trials in order to exclude the possibility that knowledge about the statements in the questionnaire (e.g., about hand ownership) could influence the pointing behavior. Half of the trials were temporarily congruent (i.e., described above), and the other half were incongruent, with the duration of each tap the same as in the congruent condition but delayed for approximately 300 ms. Additionally, in the incongruent control condition, the taps were applied alternately to the knuckle of the index and middle fingers on the real hand in order to maximize the incongruency of the stimulation and thereby eliminate the illusion as effectively as possible. If the participant affirmed the ownership of the rubber hand, that is, his or her report was equal to or greater than +1 for the question “It felt as if I was touching my right hand with my left index finger” in the congruent condition, one additional trial was conducted to estimate the onset of the illusion for purely descriptive purposes^24^. During the proprioceptive drift trials, the experimenter placed the participant’s left hand on a smooth-surfaced ruler glued to the table 10 centimeters above the rubber hand. The starting location was randomized between 40 and 60 centimeters from the location of the right hand. Participants slid their left index finger along the ruler towards the right until they perceived that it was directly above their right index finger. The procedure was repeated before and after each stimulation period (pre-trial and post-trial measures as described in^25^). The proprioceptive drift score was calculated as the difference between the pre-trial and post-trial finger localization measures. We also calculated the so-called ‘proprioceptive shift’ as the difference in proprioceptive drift between the congruent and incongruent conditions in order to examine changes in proprioceptive drift that were specific to the illusion^4,33^. Subsequently, and after the all six drift trials had been completed, by the end of each of the next two trials, participants were asked to complete a verbally administered (read by the experimenter) questionnaire regarding their experiences during the most recent trial. The questionnaire consisted of five statements from the article by Ehrsson *et al*.^25^; one was the ownership statement (Q1: “It felt as if I was touching my right hand with my left index finger”), and the four remaining questions attempted to capture a possible expectancy and task-compliance effect (Q2: “It felt like I had more than one right hand”, Q3: “It felt like my right hand was larger than normal”, Q4: “It felt like my right hand was moving”, Q5: “It seemed like I was not able to feel my own right hand”). Participants verbally rated each of the statements on a seven-point scale from −3 to +3, where −3 meant “I disagree very strongly”, +3 meant “I agree very strongly”, and 0 meant “I am uncertain”. During the final extra trial measuring the onset of the illusion, participants were asked to verbally report the feeling of ownership by saying “now” as soon as they started to feel like they were touching their right hand with their left index finger.

### Data analysis

The proprioceptive drift data were normally distributed (Shapiro-Wilk test value always >0.05). We analyzed these data by comparing the groups using two-tailed independent-samples t-tests. The proprioceptive shift data were not distributed normally (Shapiro-Wilk test value = 0.013 in the control group), so these data were analyzed with the Mann–Whitney U test. Due to being on an ordinal scale, the questionnaire data were tested non-parametrically with the Mann–Whitney U test or the Wilcoxon signed-rank test. Correlations between proprioceptive drift and the ownership statement, and between proprioceptive shift and the difference in illusion statements between the congruent and incongruent conditions were tested with the non-parametric Spearman’s rank correlation. Two-tailed tests were used in all statistical analyses.

### Data availability

All data generated and analyzed during the study are available from the corresponding author on request.

## Results

**Figure 3:**
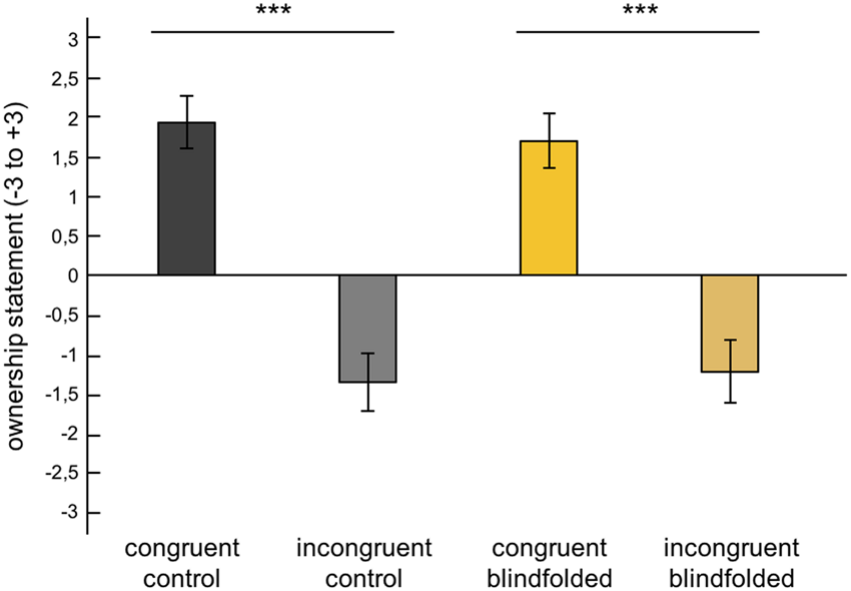
The subjective report of the illusion. Questionnaire results for the congruent and incongruent conditions. Error bars represent standard errors of the mean. Asterisks indicate a significant difference between conditions (***p < 0.001). Please note that we used non-parametric statistics but chose to display the means in order to allow better comparison with previous studies refs^27,34^).

For the questionnaire results, participants from both the blindfolded and control groups gave high affirmative ownership ratings for the congruent condition (mean ownership rating = 1.7 [SE = 0.346] and 1.93 [SE = 0.335], respectively) (Fig. 3). In contrast to our hypothesis, there was no significant difference in ratings between the groups in the congruent condition (Z = −0.271, p = 0.787), which suggests that there is no effect of short-term visual deprivation on the feeling of ownership of the rubber hand. We also performed a Bayesian t-test (BayesFactor package 0.9.12 for R) in order to test whether our data provide evidence for the absence of an effect, thus supporting the null hypothesis. Bayesian analysis revealed a Bayes factor of 3.5 in favor of the null hypothesis, indicating that the data were 3.5 times more likely under the null hypothesis than under the alternative hypothesis.

Participants in both groups also rejected the ownership of the hand in the incongruent condition and gave significantly lower scores compared to the congruent condition, which reproduced the effect from the experiment by Ehrsson *et al.*^25^ (−1.23 [SE = 0.4], Z = −4.214, p < 0.001 in the blindfolded group and −1.36 [SE = 0.372], Z = −4.318, p < 0.001 in the control group). Furthermore, there was no difference in the rejection of the control statements between the groups (−1.64 [SE = 0.244] in the blindfolded group and −1.3 [SE = 0.222] in the control group for the congruent condition [Z = −1.168, p = 0.243], and −1.64 [SE = 0.28] in the blindfolded group and −1.675 [SE = 0.195] in the control group for the incongruent condition [Z = −0.458, p = 0.647]).

**Figure 4:**
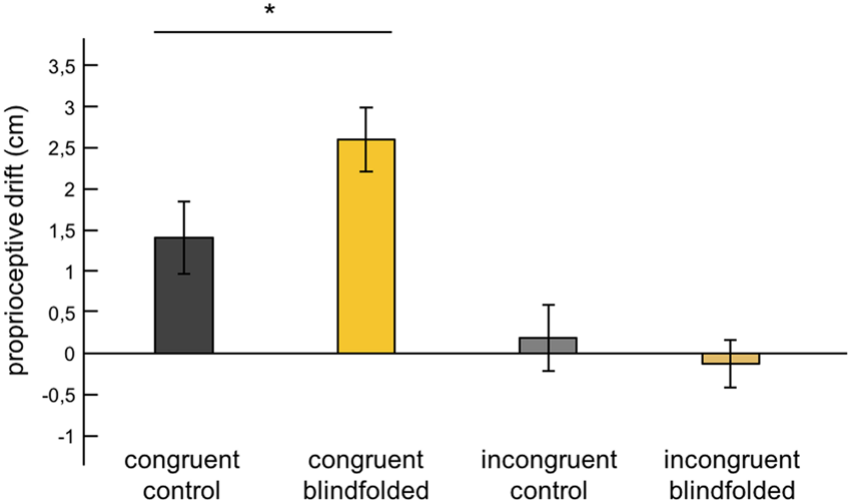
Proprioceptive drift. Average proprioceptive drift data for the congruent and incongruent conditions. Error bars represent standard errors of the mean. Asterisks indicate a significant difference between groups (*p < 0.05).

Surprisingly, proprioceptive drift was significantly greater in the blindfolded group compared to the control group, with 2.594 cm [SE = 0.385] and 1.406 cm [SE = 0.436], respectively (t(58) = 2.042, p = 0.046, CI95% = 0.024–2.354), which suggests a positive effect of short-term visual deprivation on the spatial recalibration of hand position sense (Fig. 4). Furthermore, the results reproduced the basic illusion-effect from the experiment by Ehrsson *et al*.^25^, because in both groups the congruent condition led to significantly greater proprioceptive drift compared with the incongruent condition (t(29) = 8.069, p < 0.001, CI95% = 2.032–3.412 in the blindfolded group and t(29) = 2.395, p = 0.023, CI95% = 0.178–2.266 in the control group). There was no difference in drift between the groups for the incongruent condition (t(58) = 0.633, p = 0.529, CI95% = −1.295–0.673).

**Figure 5:**
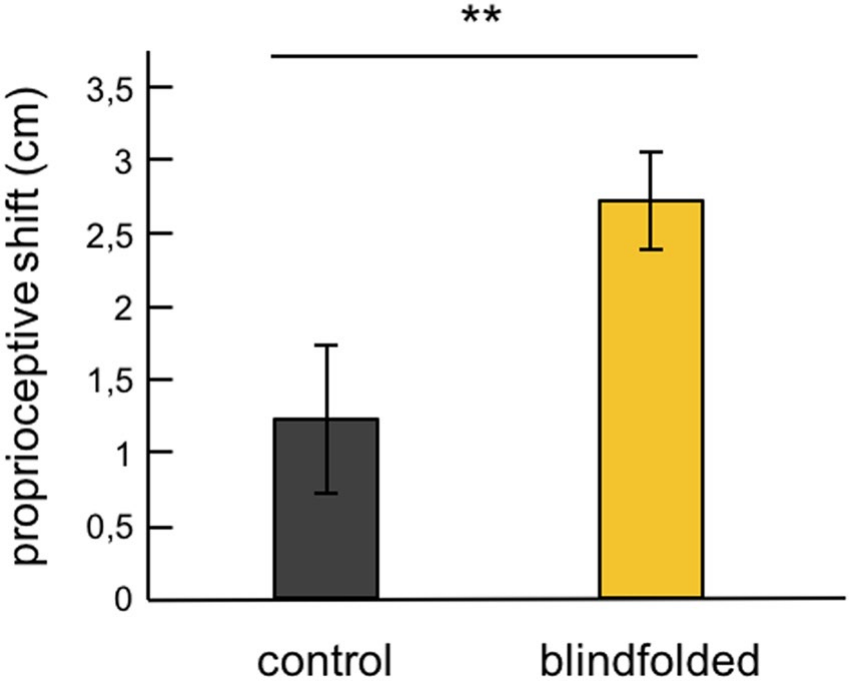
Proprioceptive shift. Average proprioceptive shift data for the control and blindfolded groups. Error bars represent standard errors of the mean. Asterisks indicate a significant difference between groups (**p < 0.01).

Moreover, a Mann–Whitney U test of proprioceptive shift, i.e., the difference in proprioceptive drift between the congruent and incongruent conditions, revealed a significantly greater proprioceptive shift in the blindfolded group compared to the control group (Z = −2.767, p = 0.006; Fig. 5). That is, the condition-specific change in proprioceptive drift towards the rubber hand was greater after blindfolding.

**Figure 6:**
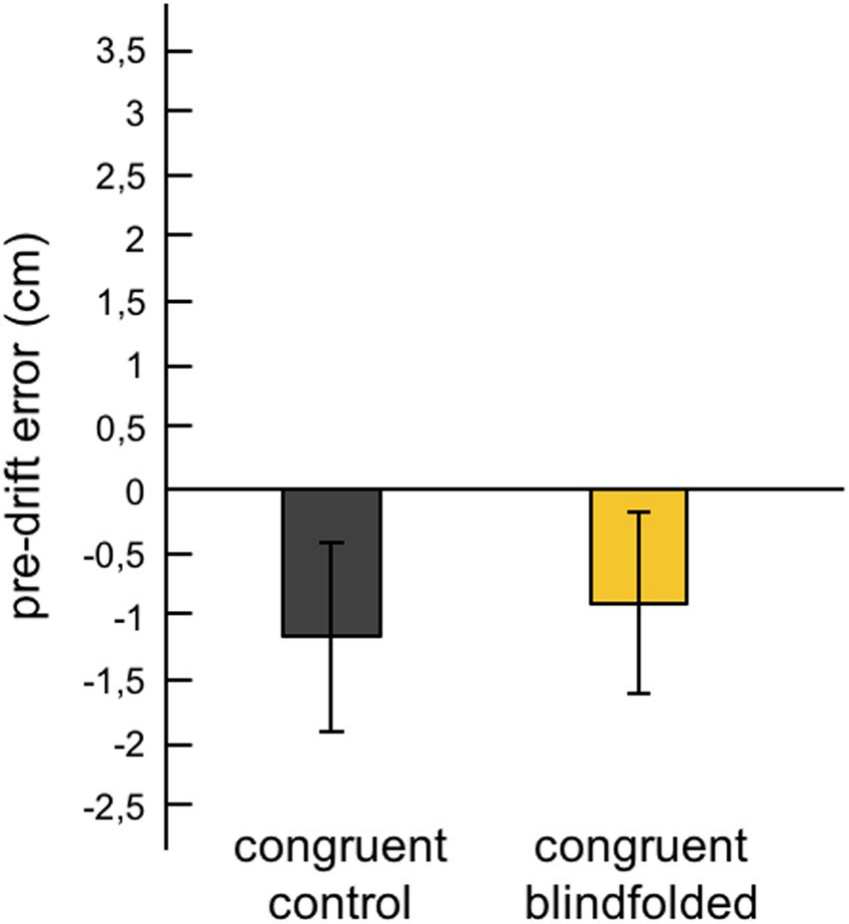
Pre-drift pointing error. Average pre-drift pointing error for the congruent condition. Error bars represent standard errors of the mean.

One concern could be that the blindfolding had a general effect on proprioception or the motor performance of the proprioceptive drift task rather than a specific effect on the spatial recalibration of hand position sense *per se*. To refute this concern, we analyzed the pointing data from the proprioceptive drift task for the pre-drift test only, which assessed the participants’ ability to indicate the perceived position of their left index finger with the right index finger without any influence of the illusion. Crucially, there was no effect of blindfolding on this proprioceptive ability – the pre-drift pointing error for the congruent condition in the blindfolded group was −0.9 cm (SE = 0.722), while in the control group, it was −1.16 cm (SE = 0.751; difference not significant – t(58) = 0.253, p = 0.801, CI95% = −1.822–2.35; Fig. 6). This finding indicates that the difference between groups in the proprioceptive drift observed in the illusion condition is not driven by general effects on proprioception or manual performance but on the spatial recalibration of hand position sense driven by the illusion.

Interestingly, in the blindfolded group, we found a significant correlation between proprioceptive drift and the ownership statement in the congruent condition (ϱ = 0.463, p = 0.01; see Supplementary Fig. 1a), in line with previous research showing a link between these two measures see^4,28,50^. This relationship was not found in the control group (ϱ = 0.055, p = 0.771). Moreover, in the blindfolded group, we found a nearly significant correlation between proprioceptive shift and the increase in illusion statement rating in congruent compared to incongruent conditions (ϱ = 0.322, p = 0.083; see Supplementary Fig. 1b); this relationship was not found in the control group (ϱ = −0.283, p = 0.129). Finally, no significant differences were found between the groups in terms of the onset times of the illusion (mean illusion onset = 11.43 s for the blindfolded group and 13.38 s for the control group; see Supplementary Fig. 2).

## Discussion

In this experiment, we studied the effect of short-term visual deprivation on the self-attribution of body parts. Contrary to our hypothesis, we found that 120 minutes of blindfolding did not have any effect on the subjective ratings of the somatic rubber hand illusion, while – remarkably – it promoted the spatial recalibration of hand position sense as evident from the significantly larger proprioceptive drift towards the rubber hand in the blindfolded group compared to the control group, but only in the congruent condition that triggered the illusion. This finding suggests that short-term visual deprivation boosts the flexibility of the body representation specifically in terms of the spatial recalibration of proprioception and touch. This finding has bearings on our understanding of how visual experience – and the lack of thereof – dynamically shapes body representation.

Therefore, what could be the mechanism behind the boosted spatial recalibration of hand position sense after the two-hour visual deprivation? Since the present study is to first to investigate the effects of blindfolding on body representation – and our findings were unexpected – our explanations will naturally be somewhat speculative. We can provide two possible explanations for the observed effects on the proprioceptive drift. First, it could be that the blindfolding caused hyper-excitability in the posterior parietal association cortex, an area that is likely to be involved in the spatial recalibration of hand position sense in the rubber hand illusion^26^. It has been shown that blindfolding leads to increased activation of the visual cortex^43,45^, which becomes engaged in processing of non-visual stimuli^36^. We also know that short-term visual deprivation is associated with increased excitability of the motor cortex^46,51^; but see also^47^. These regions are anatomically connected to the posterior parietal cortex, which has the capacity to integrate visual, tactile and proprioceptive information from the upper limb^3,12,27,52,53^ and tactile and proprioceptive signals from the two hands in the somatic rubber hand illusion^25^. Furthermore, it has been shown that changes in the activation in posterior parietal cortex correlate with proprioceptive drift^26^. Therefore, we speculate that it might be that the reduced intra-cortical inhibition leads to excitation of the posterior parietal cortex see^54,55^ to create enhanced proprioceptive recalibration during the somatic rubber hand illusion, i.e., increased processing and integration of tactile and proprioceptive signals in external body-centered spatial reference frames from the two hands^5^. This explanation would fit with that propositions of Pascual-Leone and Hamilton^56^ and Merabet *et al.*^36^ who suggested that normally inhibited or “masked” functions in the sighted can be revealed by short-term visual deprivation.

A second possible mechanism would be that the blindfolding changes how the brain weights touch and proprioception from the two hands in the dynamic illusion process. According to contemporary models of multisensory integration (i.e., maximum likelihood estimation; see^57^, the brain weights the information from the different senses according to the reliability (lowest variability) in the process of forming coherent multisensory percepts. The somatic rubber hand illusion is a consequence of integration of tactile and proprioceptive signals from the touching left index finger and tactile information from the receiving right hand into a single coherent multisensory event of bimanual self-touch. The integration of the tactile signals from the dynamic touch events on the two hands “wins” over the static proprioception sense, leading to spatial recalibration of the right hand position sense. Thus, according to this multisensory integration account, a stronger illusion could come about from stronger reliance on touch over proprioception. Therefore, the greater proprioceptive drift we observed in the blindfolded group could be explained in terms of the short-term visual deprivation enhancing the reliance of touch over other the senses, including proprioception. This interpretation would be consistent with previous studies that have shown that short-term visual deprivation can enhance tactile processing in various tasks^37,38^ but see also^39,40^, including Braille reading^58^. However, according to this explanation, the illusion should be generally stronger including its subjective component measured by the questionnaires, and not only in terms of the proprioceptive drift as we observed. However, the subjective illusion was already very high in our study, so a ceiling effect might be at play. Furthermore, the questionnaire ratings with only three levels of affirmative responses are probably less sensitive than the proprioceptive drift test. Thus, we still consider blindfolding-induced tactile-proprioceptive re-weighing as an interesting possible explanation for our results, and one that is not mutually exclusive with the idea of hyper-excitability of the posterior parietal cortex.

The present results also suggest that the absence of visual input for two hours is different from acquired or congenital blindness. We know from previous experiments with the somatic rubber hand illusion^34,35^ that both congenitally blind and late-blind individuals do not show any signs of being able to subjectively experience the illusion, while the late-blind seem to have acquired enough visual information to show spatial recalibration of the hand. In short, blind subjects show reduced illusion. Still, our results suggest that blindfolded, seeing participants show the opposite effect. One could even say that blindfolding made the seeing subjects “anti-blind” or “hyper-seeing”. Thus, our results are opposite to those of blind individuals, which raises the question of the validity of blindfolding as a model system for blindness and begs the question of whether different kinds of plasticity mechanisms are involved. Indeed, as reported by del Mar Quiroga and colleagues^59^, plasticity does not necessarily explain short-term sensory adaptation, which rather emerges from the dynamics of a recurrently connected network of neurons. Thus, 120 minutes of blindfolding was not sufficient to significantly impair multisensory integration of bodily signals or the external spatial reference frame used in this process.

The lack of effect of visual deprivation on the ownership statements together with the significant effect on the objective measure suggests that proprioceptive drift and the subjective report represent distinct aspects of the rubber hand illusion^11,31^. According to Botvinick and Cohen^1^, proprioceptive drift in the rubber hand illusion arises from a three-way interaction between vision, touch and proprioception and “the illusion’s spurious reconciliation of visual and tactile inputs relies upon a distortion of position sense” (pp. 756), arguing that both the feeling of ownership and proprioceptive drift have their roots in the same multisensory mechanism. However, there is a growing body of evidence showing that subjective illusion and proprioceptive drift sometimes do not go hand in hand, which rather suggests that these reflect two different components of the internal representations of the body. The first, related to body ownership, reflects more the conscious experience of one’s own body as a coherent multisensory percept^7^, and the second, related to proprioceptive drift, is a less conscious, constantly updated model of one’s own body based on past sensory experiences (proprioceptive, tactile and vestibular) involved in action planning and action control^60,61^. In contrast to the assumption that body ownership can be more or less equated with proprioceptive drift^4^, our results show that under the specific circumstance of two-hour visual deprivation, one of the components of the rubber hand illusion – proprioceptive drift – can be significantly boosted, while another one – subjective rating of the illusion – remains unchanged. Thus, our results add to the growing literature of studies that indicate that subjective body ownership and proprioceptive drift should be treated as two distinct processes, even if they are closely related and sometimes highly correlated^11,31–33^.

Taken together, our results show that short-term visual deprivation leads to increases in the plasticity of body representation in terms of the spatial recalibration of proprioception and touch from the two upper limbs. This interesting and unexpected observation shows that short-term visual deprivation affects not only specific single modalities as we know from previous studies but also complex multisensory interactions related to the representation of one’s own physical self. Additionally, our results provide evidence for a dissociation of the processes related to proprioceptive drift and multisensory ownership perceptions since only the former was affected by the blindfolding procedure.

## Electronic supplementary material

Supplementary Figures
