# Supplementary material for: Short-term visual deprivation boosts the flexibility of body representation
Source: Sci Rep. 2018 Apr 19;8:6284. doi: 10.1038/s41598-018-24496-8 (PMC5908916; doi:10.1038/s41598-018-24496-8)

Short-term visual deprivation boosts the flexibility of body representation

Dominika Radziun[1*], H. Henrik Ehrsson[1*]

[1] Department of Neuroscience, Karolinska Institutet, Stockholm, Sweden

*E-mail: nika.radziun@gmail.com (DR); henrik.ehrsson@ki.se (HHE)

Supplementary Fig. 1a

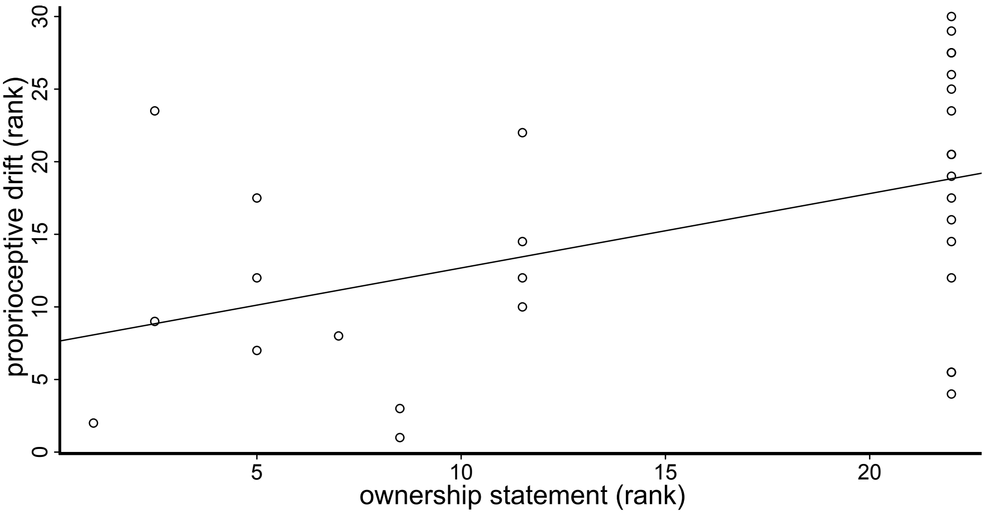

Supplementary Figure 1a. Correlation between proprioceptive drift and the ownership statement in the congruent condition in the blindfolded group.

Supplementary Fig. 1b

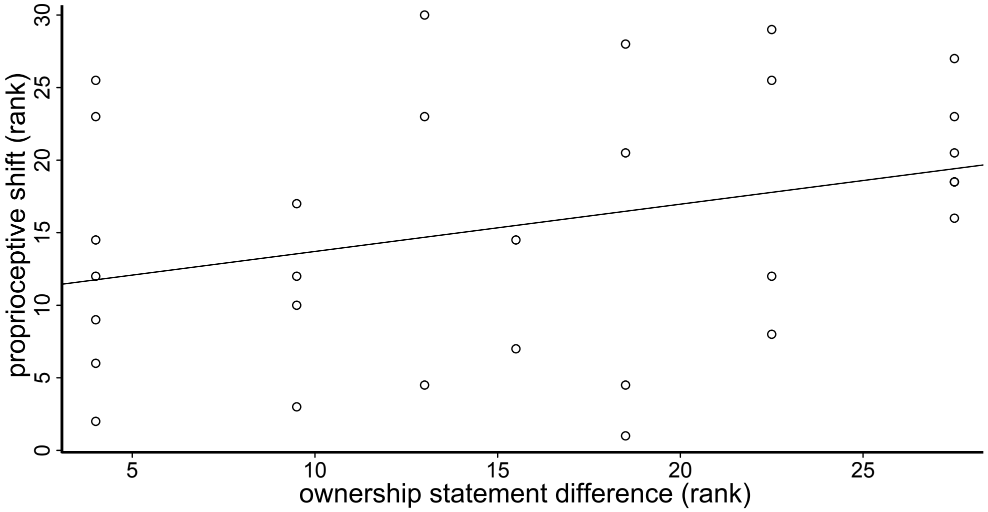

Supplementary Figure 1b. Correlation between proprioceptive shift and the ownership statement difference between the congruent and incongruent conditions in the blindfolded group.

Supplementary Fig. 2

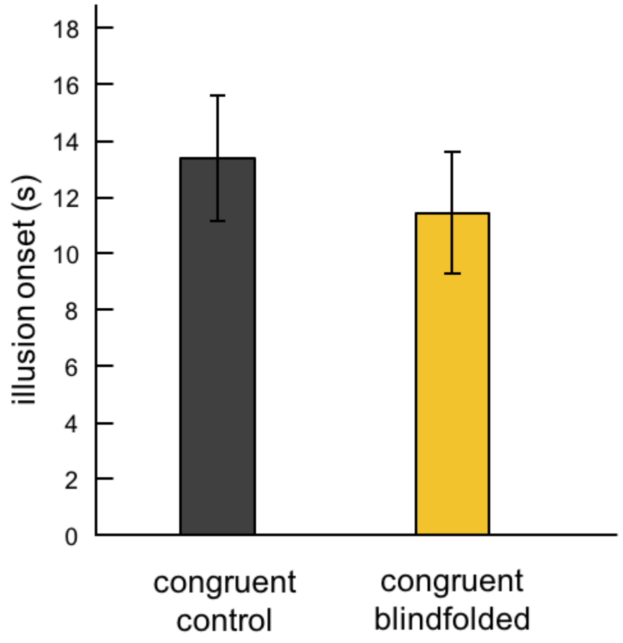

Supplement: Supplementary file 1 — Supplementary Figures [file 41598_2018_24496_MOESM1_ESM.pdf]
